# Genome-wide analysis of MYB transcription factors and their responses to salt stress in *Casuarina equisetifolia*

**DOI:** 10.1186/s12870-021-03083-6

**Published:** 2021-07-08

**Authors:** Yujiao Wang, Yong Zhang, Chunjie Fan, Yongcheng Wei, Jingxiang Meng, Zhen Li, Chonglu Zhong

**Affiliations:** Research Institute of Tropical Forestry, Chinese Academy of Forestry, Guangzhou, 510520 China

**Keywords:** *Casuarina equisetifolia*, *MYB* transcription factor, GO annotation analysis, Salt stress

## Abstract

**Background:**

*MYB* transcription factors are a kind of DNA binding protein that can specifically interact with the promoter region. Members of *MYB* TFs are widely involved in plant growth and development, secondary metabolism, stress response, and hormone signal transduction. However, there is no report of comprehensive bioinformatics analysis on the MYB family of *Casuarina equisetifolia*.

**Results:**

In this study, bioinformatics methods were used to screen out 182 *MYB* transcription factors from the *Casuarina equisetifolia* genome database, including 69 1R-MYB, 107 R2R3-MYB, 4 R1R2R3-MYB, and 2 4R-MYB. The *C. equisetifolia* R2R3-MYB genes were divided into 29 groups based on the phylogenetic topology and the classification of the *MYB* superfamily in *Arabidopsis thaliana*, while the remaining *MYB* genes (1R-MYB, R1R2R3-MYB, and 4R-MYB) was divided into 19 groups. Moreover, the conserved motif and gene structure analysis shown that the members of the *CeqMYB*s were divided into the same subgroups with mostly similar gene structures. In addition, many conserved amino acids in the R2 and R3 domains of *CeqMYB*s by WebLogo analysis, especially tryptophan residues (W), with 3 conserved W in R2 repeat and 2 conserved W in R3 repeat. Combining promoter and GO annotation analysis, speculated on the various biological functions of *CeqMYB*s, thus 32 *MYB* genes were selected to further explore its response to salt stress by using qPCR analysis technique. Most *CeqMYB* genes were differentially regulated following multiple salt treatments.

**Conclusions:**

Seven genes (*CeqMYB164*, *CeqMYB4*, *CeqMYB53*, *CeqMYB32*, *CeqMYB114, CeqMYB71* and *CeqMYB177*) were assigned to the “response to salt stress” by GO annotation. Among them, the expression level of CeqMYB4 was up-regulated under various salt treatments, indicating *CeqMYB4* might participated in the response to salt stress. Our results provide important information for the biological function of *C. equisetifolia*, as well as offer candidate genes for further study of salt stress mechanism.

**Supplementary Information:**

The online version contains supplementary material available at 10.1186/s12870-021-03083-6.

## Background

*MYB* (v-myb avian myeloblastosis viral oncogene homolog) transcription factor (TF) family is one of the largest transcription factors in plants and gets its name because its structure has a conserved DNA binding region, known as MYB domain. The N-terminal regions of the *MYB* transcription factor domain are highly conserved, and the domain consists of 1–4 serial and nonredundant imperfect sequence repeats (R1, R2, R3 and R4). Each repeat includes about 50 amino acids, containing a series of highly conserved amino acid residues and interval sequences, wherein the amino acid residues participate in the binding process with DNA in the form of helix-turn-helix (HTH) [1, 2]. MYB proteins can be divided into different classes according to the number of repeats: 1R-MYB (one repeats), R2R3-MYB (two repeats), R1R2R3-MYB (three repeats), and 4R-MYB (four repeats) [3, 4].

As regulatory protein, *MYB* TFs play an important role in plant growth and development. In 1987, Paz-Ares et al. identified the first plant *MYB* gene in maize, and the research showed that it was related to anthocyanin synthesis, and named it *ZmMYBC1* [5]. Since then, the *MYB* gene had been identified and isolated in many species. Among them, R2R3-MYB TFs had been certified to be widely involved in the regulation of plant secondary metabolism, and acted a key role in the regulation of plant cell differentiation and organ formation [4, 6, 7]. Overexpression of *MYB6* in transgenic poplar resulted in significantly increased anthocyanin and procyanidins accumulation, but inhibited the development of secondary cell walls [8]. And Mu et al. found that R2R3 TF *AtMYB59* could regulate the cell cycle and root growth of *Arabidopsis thaliana* [9] and JcMYB1 (R2R3-MYB) played an important role in the abiotic stress response [10]. In addition, *MYB30* in defense reaction was important for the regulation of root growth, and *LcMYB2* promoted seed germination and root growth under drought stress [11]. It was found that 3R type MYB *MYBL2* not only inhibited trichome development, but also inhibited flavonoid biosynthetic [12, 13], and 3R type MYB *PhMYBx* down-regulated anthocyanin synthesis [14, 15]. Previous study had found that the expression of *AtMYB2* was up-regulated in late plant development and participated in the regulation of whole plant senescence [16].

*MYB* family genes are also widely involved in plant responses to hormones and environmental factors, and play an important regulatory role in plant responses to stress. For example, the R2R2-MYB TF *AtMYB41* could not only affect cell wall development, but also respond to ABA, drought, and salt stress [17]. Previous studies showed that *AtMYB96* enhanced the resistance of *A. thaliana* to low temperature by promoting the expression of *CBF*s [18], while *AtMYB14* and *AtMYB15* participated in low-temperature response by negatively regulating the expression of *CBF*s [19, 20]. What’s more, *AtMYB60* in *A. thaliana* had been shown to be involved in drought tolerance stress in plants [21]. Recently, *ZmMYB3R* and *GmMYB118* were found that could improve tolerance to drought and salt stress in transgenic plants [22, 23]. Additionally, *AtMYB2*, *AtMYB44* and *AtMYB74* enhanced the tolerance of *A. thaliana* to salt stress [24–26], while *AtMYB73* played a negative role in plant salt stress resistance [27]. Furthermore, *TaMYB73*, *StMYB30* and *GhMYB73* were reported to enhance salt stress tolerance in transgenic plants [28–30]. In transgenic tomato plants, *SlMYB102* increased the salt tolerance by regulating Na^+^-K^+^ homeostasis and ROS balance [31]. Although most *MYB* genes in response to salt stress belonged to R2R3 type, a few 3R-MYB genes (including *OsMYB3R-2* and *TaMYB3R1*) were involved in the regulation of plant salt stress [32, 33].

The Casuarinaceae is a relatively special family of angiosperms, and is relatively distantly related to other plants [34]. *C. equisetifolia* is widely cultivated in tropical and subtropical regions and have many uses. It is suitable for coastal windbreak and sand fixation, saline-alkali land improvement and afforestation in arid areas. It can also fix nitrogen and provide wood and fuelwood, and is applied in agroforestry [35]. In addition, Casuarina is one of the few plants that thrive on coastal beaches because of its salt resistance and the flexibility of its branches that can withstand typhoons. The availability of the complete Casuarina sequence [36] combined with bioinformatics methods provides an opportunity to conduct a comprehensive, genome-wide analysis of *C. equisetifolia MYB* genes.

The *MYB* gene family has been extensively studied in monocot and dicot plant. However, current basic knowledge of MYB proteins in *C. equisetifolia* is still limited. In present study, a total of 182 *MYB* genes were identified using the known *MYB* gene sequences from *A. thaliana* genome. Furthermore, the physical position, phylogenetic relationships, conserved motifs and exon-intron structure were performed. We further analyzed selective pressure, cis-acting element and gene ontology of these genes. Finally, the expression levels of selected *CeqMYB* genes in roots and shoots under different salt concentrations and treatment times were investigated by using RNA-seq data and qRT-PCR. Based on current data, this is the first report on genome-wide gene family identification in *C. equisetifolia*. Therefore, the present study provides a starting point to explore the functions of *MYB* genes in *C. equisetifolia*, and it also helps to select candidate genes for genetic engineering in *C. equisetifolia* breeding.

## Results

### Identification of MYB genes in *C. equisetifolia*

The candidate genes with typical MYB or MYB-like domains were preliminarily screened from *Casuarina* genomic database according to the Hidden Markov Model (HMM) profile of the MYB domain. A total of 182 *MYB* genes were identified in *C. equisetifolia* after removing redundant repetitive sequences. Based on the order of gene identifier, *CeqMYB* genes were named *CeqMYB1* to *CeqMYB182*. The lengths of the protein sequences of CeqMYB range from 104 to 1072 amino acids, and molecular weight vary from 11.08 kDa (*CeqMYB153*) to 117.93 kDa (*CeqMYB158*). Moreover, the theoretical isoelectric point (pI) ranged from 4.06 to 10.65. Some other parameters, such as scaffold position, open reading frame (ORF) length and number of domains, were detailed in the Table S1. The predicted subcellular localization data (Table S2) showed that most CeqMYB proteins were predicted to be expressed in the nucleus, while some were localized to chloroplasts (CeqMYB41, CeqMYB114 and CeqMYB148), mitochondria (CeqMYB37), and cytoplasm (CeqMYB5 and CeqMYB23).

### Phylogenetic trees and group classification of *CeqMYB* genes

**Fig. 1:**
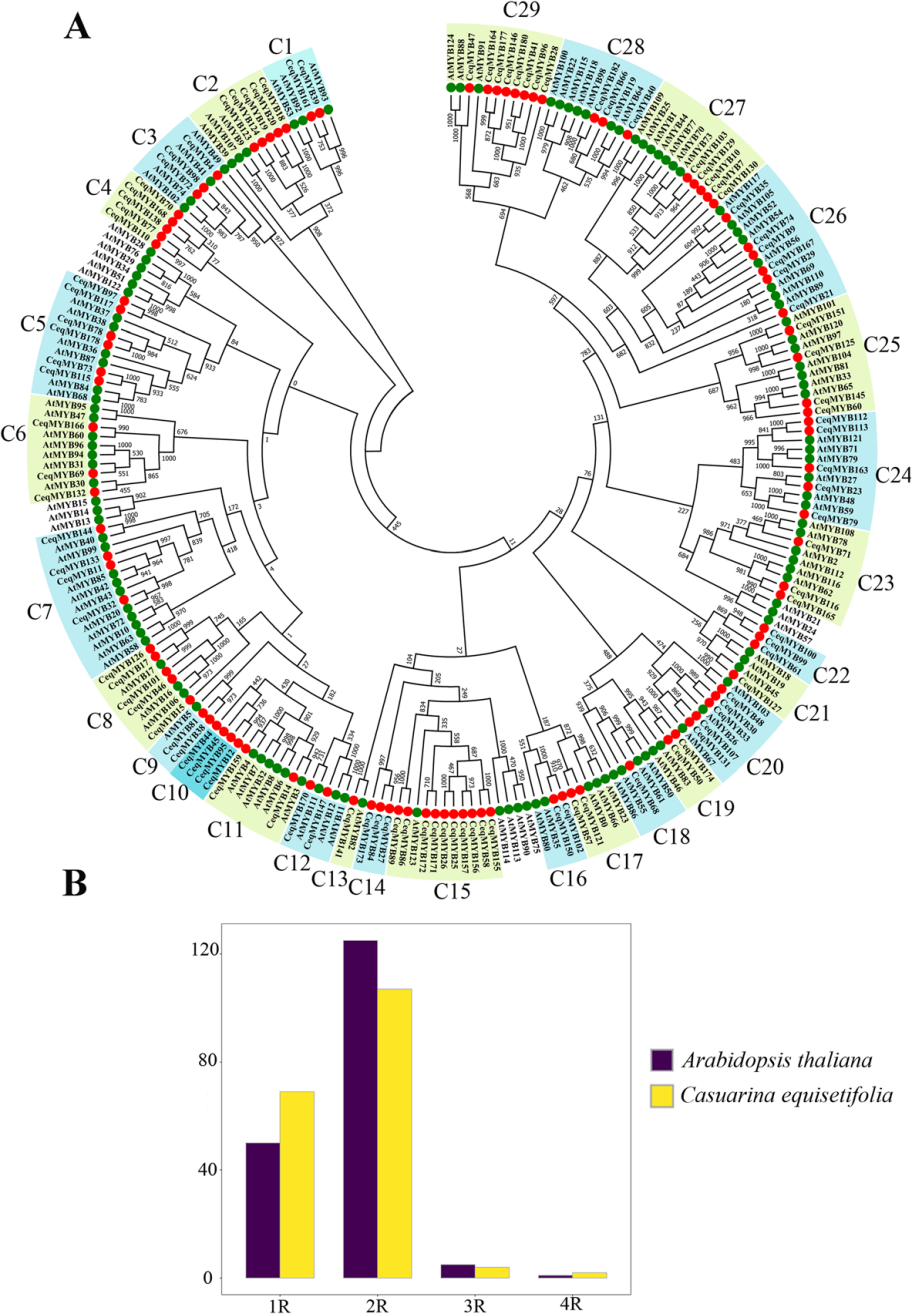
Phylogenetic relationships among R2R3-MYB genes in *Casuarina equisetifolia* and *Arabidopsis thaliana.* **A** Putative functions of the MYB proteins in *C. equisetifolia* based on the phylogenetic tree along with MYBs from *A. thaliana*. For details of the functions see Table S3. The circular unrooted tree was generated by NJ method with 1000 bootstrap replicates. **B** The number distribution of MYB gene in *C. equisetifolia* and *A. thaliana*

**Fig. 2:**
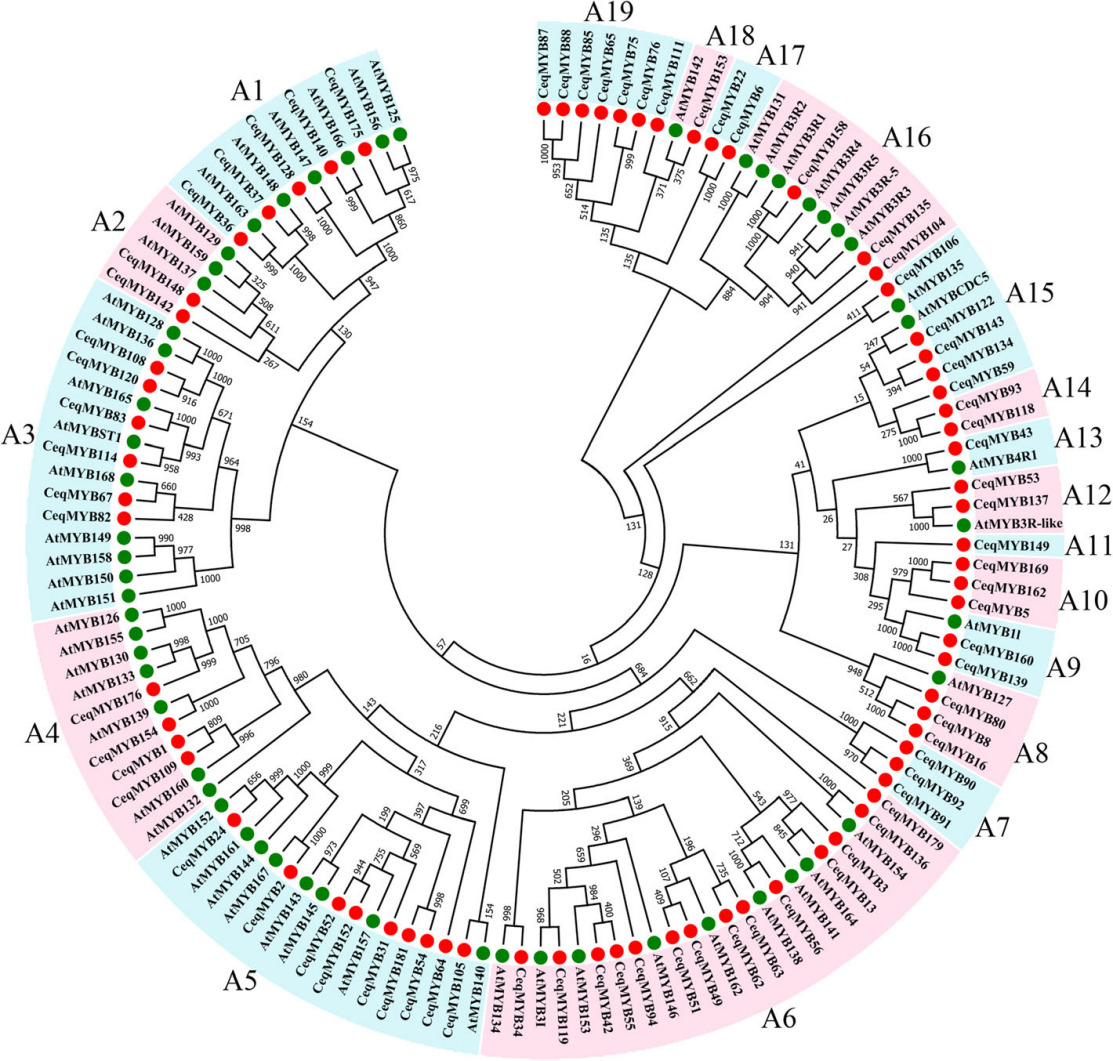
Phylogenetic tree of 1R-MYB, 3R-MYB and 4R-MYB genes in *Casuarina equisetifolia* and *Arabidopsis thaliana*. The circular unrooted tree was generated by NJ method with 1000 bootstrap replicates

A total of 69 1R-MYB proteins, 107 R2R3-MYB proteins, four R1R2R3-MYB proteins, and two 4R-MYB proteins were identified in *C. equisetifolia* (Table S1). Based on the alignment, two phylogenetic trees were constructed using MYB proteins in *A. thaliana* and *C. equisetifolia* (Figs. 1A and Fig. 2). The number distribution of *MYB* gene in *C. equisetifolia* was consistent with that in *A. thaliana* (Fig. 1B). R2R3-MYB protein was the largest subfamily, while the number of 4R-MYB subfamily was the smallest.

The first phylogenetic tree (Fig. 1A) contained 125 R2R3-MYB genes of *A. thaliana* and 107 R2R3-MYB genes of *C. equisetifolia*. Then, these 107 R2R3-MYB genes in *C. equisetifolia* were divided into 29 groups (C1 ~ C29) according to the topology of the tree and the classification of the *MYB* superfamily in *A. thaliana* (Table S3). C13, C18 and C21 had only 1 member, which was the smallest group, while C15 had 10 members, which was the largest group. In addition, the members of C4, C10, C14, and C22 did not cluster into any group of *A. thaliana*, indicating that some changes occurred among *MYB* genes of different species during the evolutionary process. Most groups in our study (C1, C3, C5, C6, etc.) were supported by the previous studies with high bootstrap. For example, *AtMYB93*, *AtMYB92*, and *AtMYB53* in group C1 regulated root development, and *AtMYB49*, *AtMYB41*, *AtMYB74*, and *AtMYB102* in group C3 responded to adversity stress.

In the second phylogenetic tree (Fig. 2), a total of 131 MYB proteins (119 1R-MYB, nine 3R-MYB and three 4R-MYB) from *C. equisetifolia* and *A. thaliana* were extracted to construct the phylogenetic tree. According to the topology of the tree and classifications in *A. thaliana*, these 131 MYB proteins were divided into 19 groups (A1 ~ A19). As shown in Fig. 2, one 4R-MYB protein (*CeqMYB43*) was clustered into the 4R-MYB protein of *A. thaliana*, three R1R2R3-MYB proteins (*CeqMYB104*, *CeqMYB135* and *CeqMYB158*) were clustered into five R1R2R3-MYB proteins of *A. thaliana*.

### *CeqMYB* genes structure and protein motif analysis

**Fig. 3:**
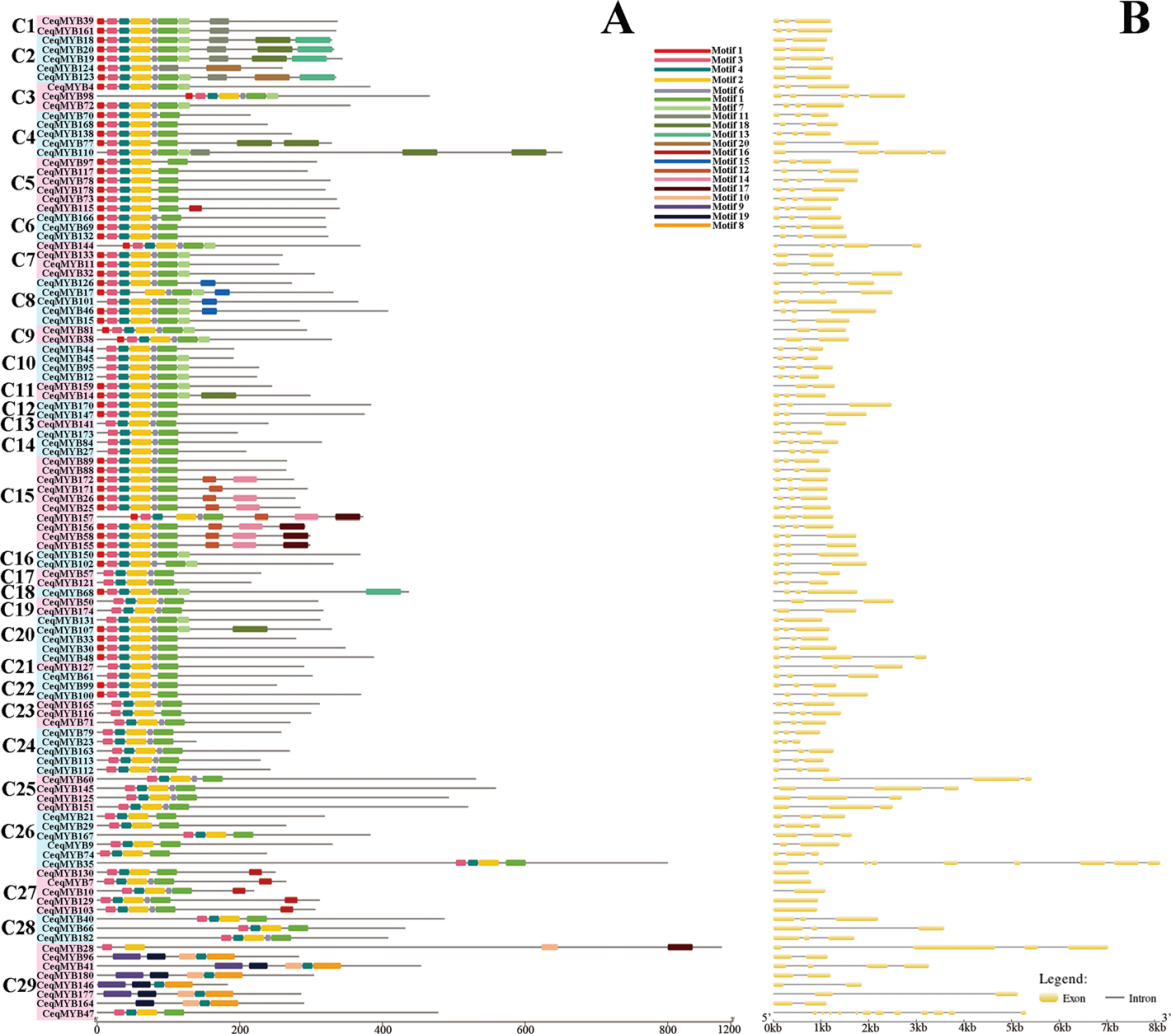
Conserved motif and gene structure analysis of the R2R3-MYB proteins in *Casuarina equisetifolia*. **A** Groups of R2R3-MYB genes are highlighted with different colored backgrounds, and all motifs were identified by MEME. The different colored boxes represent different motifs and their position in each MYB sequence. For details of the motifs see Table S4. **B** The exons and introns are indicated by yellow rectangles and black lines, respectively

**Fig. 4:**
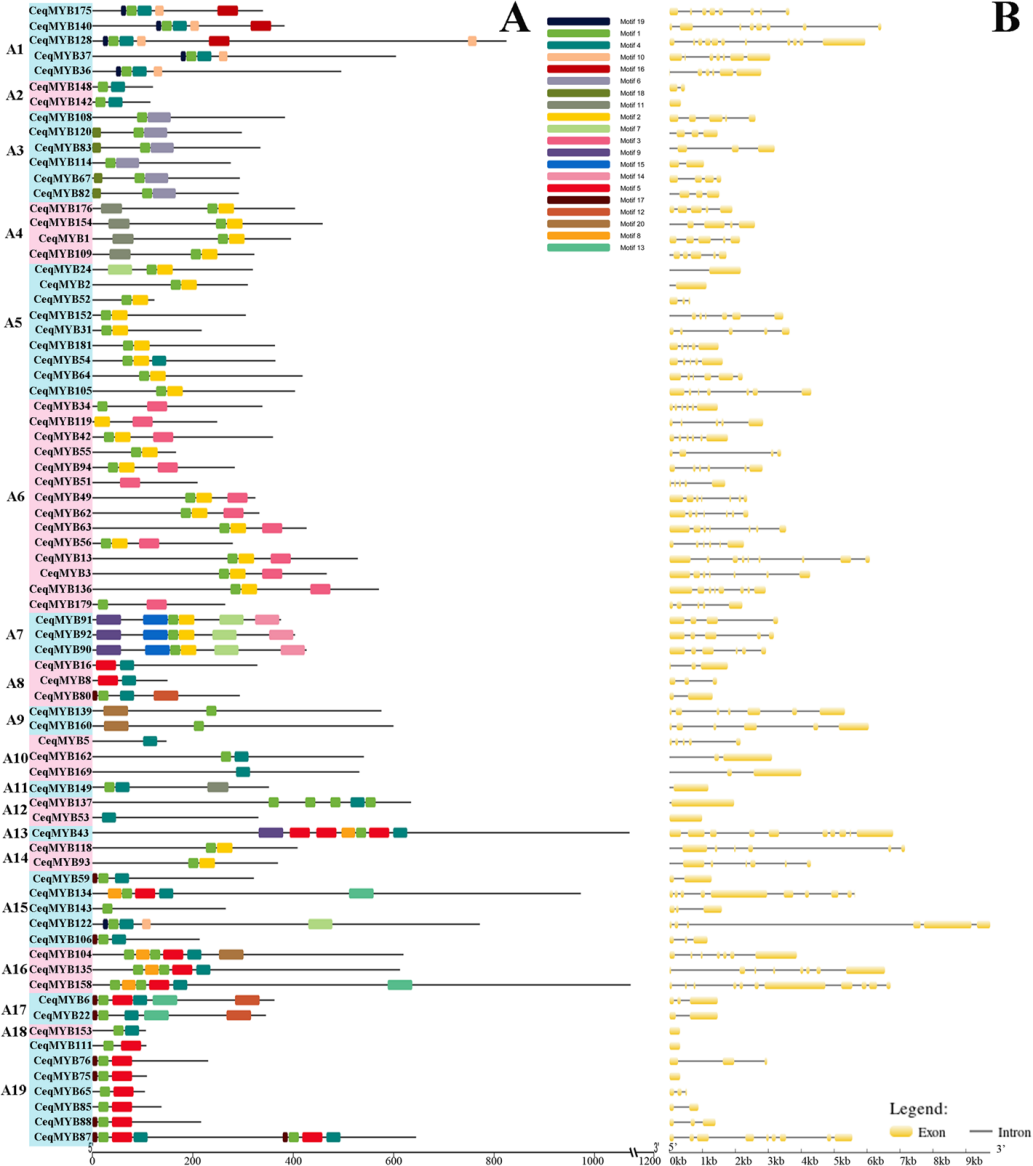
Distributions of conserved motifs gene structure and in *CeqMYB* genes (1R-MYB, 3R-MYB and 4R-MYB). **A** The motifs of numbers 1–20 is indicated in different colored boxes. The sequence information of the motifs is provided in Table S5. **B** The yellow boxes and black lines indicate exons and introns, respectively

In order to have a more comprehensive understanding of the conserved domains of the *CeqMYB* genes, The Motif Elicitation (MEME) analysis was performed. Twenty conserved motifs were found in the R2R3-MYB and other *CeqMYB*s (1R-MYB, 3R-MYB and 4R-MYB) proteins of *C. equisetifolia* (Figs. 3A; Fig. 4A). As shown in Fig. 3A and Table S4, motif 1, motif 2, motif 3, motif 4, motif 9 and motif 10 were found to encode the MYB DNA-binding domain, while the other motifs didn’t have function annotation in R2R3-MYB of *C. equisetifolia*. A total of 100 of the 107 R2R3-MYB proteins included motif 3, motif 4, motif 2, motif 6 and motif 1. *CeqMYB28* was composed of motif 3 and motif 2, motif 10 and motif 17. *CeqMYB96*, *CeqMYB41*, *CeqMYB180*, *CeqMYB146*, *CeqMYB177* and *CeqMYB164* clustered in the C29 group had unique motifs, including 9, 19, 10, 4 and 8 in R2R3-MYB proteins of *C. equisetifolia*. This characteristic was similar to a previous study in Chinese jujube (*Ziziphus jujuba Mill*.) R2R3-MYB proteins [3]. In addition, the conserved motif of other *CeqMYB* genes (1R-MYB, 3R-MYB and 4R-MYB) were predicted, different groups had different motifs, and motif 1 was common to all *MYB* genes in Fig. 4A. Specifically, motif 1 repeated four times in *CeqMYB137*, but repeated twice in members of A16 group. And motif 5 repeated three times in *CeqMYB43*. The motifs 17,1,5, and 4 were each repeated twice in *CeqMYB87*. The similar result was also found in *Helianthus annuus L* [37]. Furthermore, the result of PFAM and SMART annotation were shown in Table S5, Motif 1, motif 2, motif 5, motif 6, and motif 8 were found to encode the MYB DNA-binding domain and motif 4 encode the MYB-CC type domain, while the other motifs didn’t have function annotation.

**Fig. 5:**
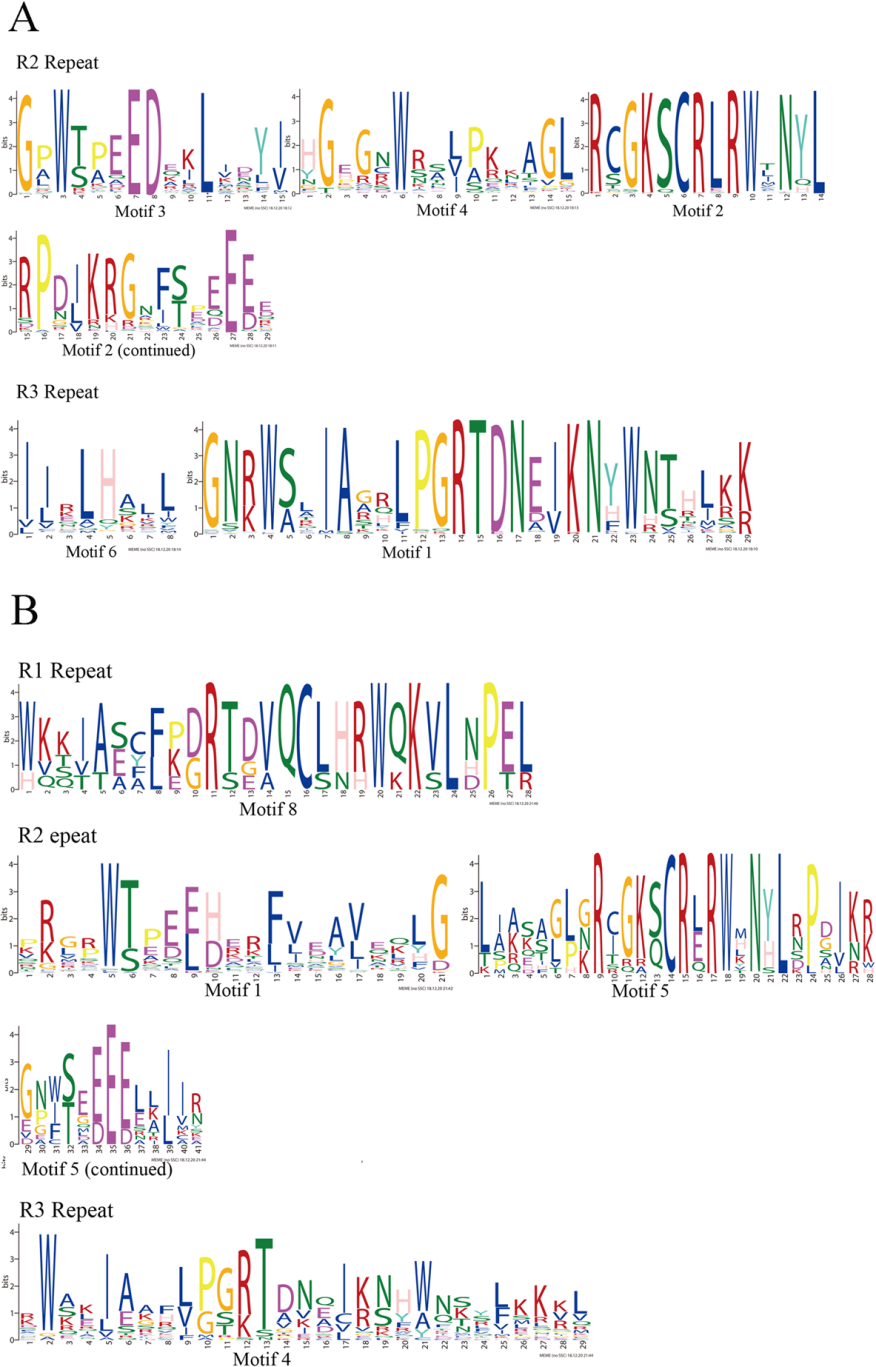
R1, R2 and R3 MYB repeats of the proteins in *CeqMYB* gene family. **A** R2 and R3 MYB repeats of R2R3-MYB proteins in *C. equisetifolia.* **B** R1, R2 and R3 MYB repeats of 1R-MYB, 3R-MYB and 4R-MYB proteins in *C. equisetifolia*. The overall height of each stack showed the conservation of the MYB protein sequence at that position. English letters indicate the different type of amino acid residue

To demonstrate conservation at particular positions, WebLogo was used to investigate further. The results (Fig. 5) showed that there were many conserved amino acids in the R2 and R3 domains of *CeqMYB*s, especially tryptophan residues (W), with 3 conserved W in R2 repeat. In the R3 repeat, there were only two conserved W residues, and the first W is usually replaced by either leucine (L) or phenylalanine (F) [38], which was replaced by L in this study.

In order to gain more insights into the structural diversity of *MYB* TFs, the exon/intron organization of R2R3-MYB and other *MYB* (1R-MYB, 3R-MYB and 4R-MYB) of *C. equisetifolia* were demonstrated separately. As shown in Fig. 3B, the 107 R2R3-MYB genes contained different numbers of exons, varying from 1 to 12. We found that most of the R2R3-MYB genes had two (20/107) or three (71/107) exons, while those with 9 and 12 exons existed just one each. In addition, combined with phylogenetic tree classification, it was found that there were 5 genes containing one exon and these 5 genes were clustered in the same group C27. Furthermore, the details of the exon/intron structural analysis of other *CeqMYB* genes (1R-MYB, 3R-MYB and 4R-MYB) were shown in Fig. 4B. The number of exons in 1R-MYBs ranged from 1 to 11, and the number of exons in 3R-MYBs ranged from 8 to 11. What’s more, the two 4R-MYBs had 9 (*CeqMYB87*) and 10 (*CeqMYB43*) exons, respectively. Based on the above observations, it was found that *CeqMYB* genes clustered in the same group had the same or similar number of exons. For example, *CeqMYB* genes belonging to the C28 (3 exons) and A14 (6 exons) group had the identical number of exons, while the A7 group had 4 to 6 exons. In brief, the number of exons of *MYB* genes from *C. equisetifolia* was quite different, but the closer the phylogenetic trees were, the greater the similarity of gene structure was.

### *MYB* paralogs and orthologs

**Fig. 6:**
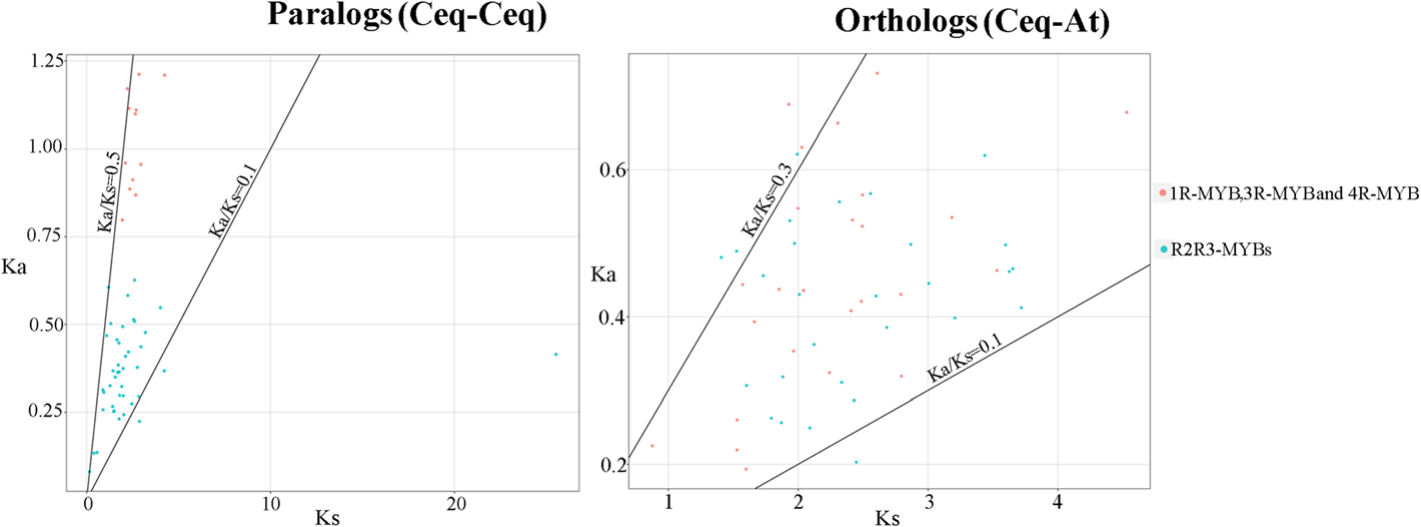
Ka/Ks ratios of paralogs and orthologs. The black lines indicated Ka/Ks equal to 0.1, 0.3 and 0.5. The red dots represented 1R-MYB, 3R-MYB and 4R-MYB genes in *C. equisetifolia* and *A. thaliana*. The green dots represented R2R3-MYB genes in *C. equisetifolia* and *A. thaliana*

A total of 53 orthologues and 54 paralogues were identified based on the topology of phylogenetic tree and BLASTN methods. The ratio of non-synonymous substitution (Ka) and synonymous substitution (Ks) can reflect the selection pressure in the process of organism evolution. Thus, to explore the role of selection pressure in *MYB* gene family evolution, Ks values, Ka values, and Ka/Ks ratios of paralogues and orthologues were obtained (Table S6 and Fig. 6). Generally, a ratio of Ka/Ks less than 1 represent purification selection; a ratio of Ka/Ks greater than 1 mean positive selection; a ratio of Ka/Ks equal to 1 indicate neutral selection. The Ka/Ks ratio of all *CeqMYB* gene pairs was less than 1, of which the Ka/Ks ratio of most orthologues was 0.1–0.3, and that of paralogues was 0.1–0.5 (Fig. 6). The result suggested that purifying selection might have played an important role in the evolution of the *MYB* genes in *C. equisetifolia*.

### Profiling of expressed *CeqMYB* genes and GO annotation analysis

**Fig. 7:**
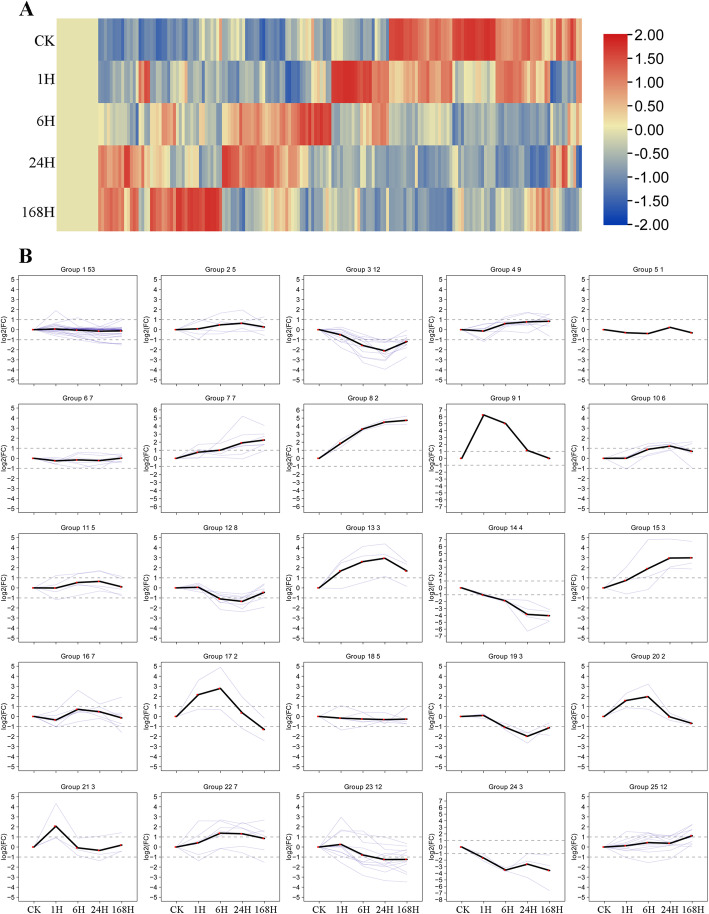
Expression pattern of 182 *CeqMYB* genes following NaCl treatment at different time points as determined by RNA-Seq. **A** The heatmap shows the hierarchical clustering of 182 *CeqMYB* genes at different time points. The color scale represents log10 expression values, blue represents low expression and red indicates a high expression level (transcript abundance). **B** Trend analysis of 182 *CeqMYB* genes expression (25 trends)

Based on transcriptome data, heat maps of *CeqMYB* genes treated with 200 mM NaCl at different time periods were analyzed (Fig. 7A). Next, the heat map data of 182 *MYB* genes were clustered into 25 expression patterns for trend analysis (Fig. 7B; Table S7). The *CeqMYB* genes responded to different time periods after 200 mM NaCl treatment, such as *CeqMYB122* and *CeqMYB14* were strongly up-regulated at 24 and 168 h after salt treatment, while the expression levels of *CeqMYB10* and *CeqMYB112* were up-regulated at 1 h after salt treatment and then down-regulated at later time points. Some of paralogues had similar expression patterns; for example, *CeqMYB163/− 113* was initially up-regulated and reached a maximum at 6 h, but then decreased gradually. Nevertheless, some of the paralogues showed different expression patterns; for instance, the expression of *CeqMYB16* was continuously up-regulated after salt treatment and reached the highest level after 168 h, while its paralogue, *CeqMYB140*, was highly expressed in 1 h during salt stress.

**Fig. 8:**
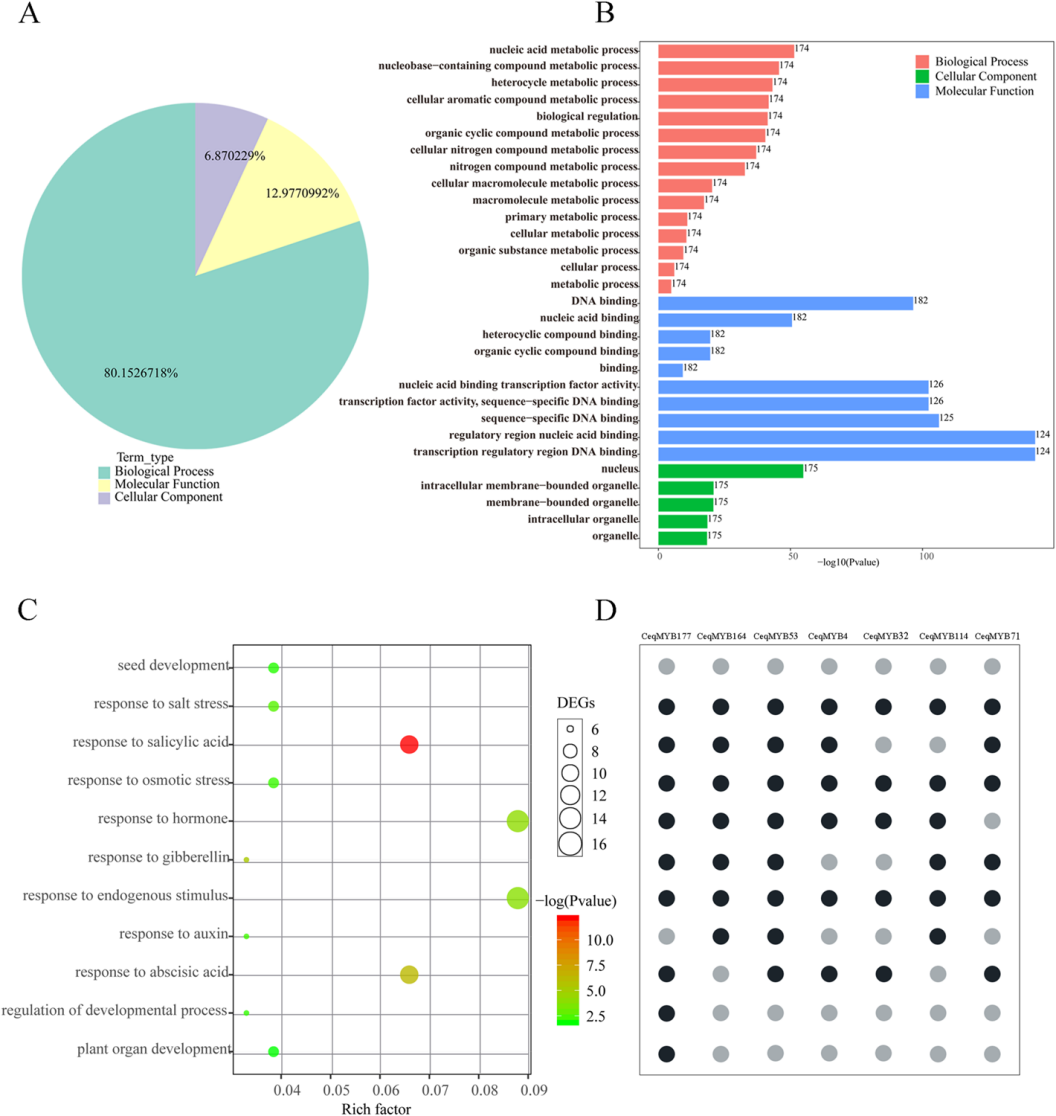
GO enrichment analysis of 182 *CeqMYB* genes under salt stress. **A** the GO annotation using a cut-off value of *P* ≤ 0.05 showed that a total of 131 GO items, including molecular function, biological process, and cellular component. **B** The numbers of predominant GO items. **C** Some *CeqMYB* genes assigned to the categories associated with development, hormone, and stress response. The color gradient represents the size of the *P* value and the size of circular represents number of *CeqMYB* genes. The “rich factor” shows the ratio of the number of the *CeqMYB* genes to the total gene number in certain categories **D** Seven genes were assigned to the “response to salt stress” category. The grey circular indicated the gene was not involved in the category. The black circular indicated the gene was assigned to relevant category

In order to predict the function of the MYB protein in *C. equisetifolia*, the GO annotation using a cut-off value of *P* ≤ 0.05 showed that a total of 131 GO items were enriched during the salt treatment (Table S8). As shown in Fig. 8A, 80% of term were categorized into biological process. Among these terms, nucleus, organelle, binding, biological regulation, cellular process and metabolic process were predominant (Fig. 8B). The analysis of cell component annotation showed that these proteins were mainly located in the nucleus, and the results were consistent with the prediction of subcellular localization. Furthermore, it was found that some *CeqMYB* genes assigned to the categories associated with development, hormone, and stress response. Seven genes were assigned to the “response to salt stress” category, of which *CeqMYB164*, *CeqMYB4*, *CeqMYB53*, *CeqMYB32*, *CeqMYB114* and *CeqMYB71* were also assigned to the categories associated with hormone response. Specially, *CeqMYB177* was in the categories associated with development, hormone, and stress response (Fig. 8C; D).

In summary, combined with the expression pattern after salt treatment and GO annotation analysis, 32 *CeqMYB* genes were selected for further analysis.

### Promoter analysis

**Fig. 9:**
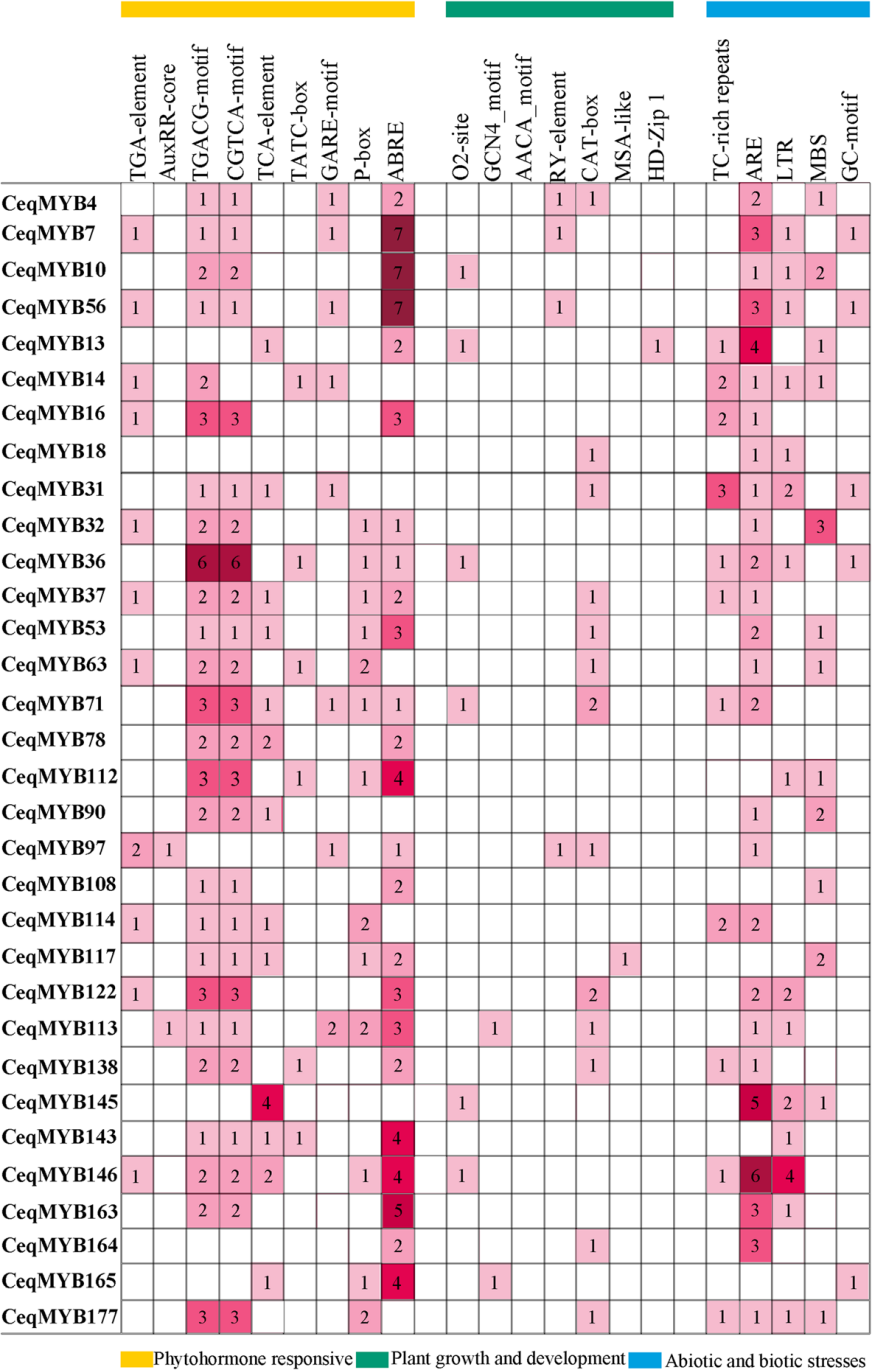
Cis-acting elements analysis of 32 selected *CeqMYB* genes in promoter region. Number of each cis-acting element in the promoter region

The cis-element analysis of 32 selected *CeqMYB* genes were shown in Fig. 9, O2-site involved in zein metabolism regulation was found in the promoters of 4 *CeqMYB* genes. The differentiation of the palisade mesophyll cells (HD-Zip 1) and cell cycle regulation (MSA-like) element were found in the *CeqMYB13* and *CeqMYB117* promoter, respectively. Additionally, the meristem expression (CAT-box), seed-specific regulation (RY-element) and endosperm expression (GCN4_motif) were also identified in the promoters of the *CeqMYB* genes. Many hormone-responsive elements were identified, the auxin-responsive element (TGA element and AuxRR-core), the SA-responsive element (TCA element), the MeJA-responsive element (CGTCA motif and TGACG motif), the gibberellin-responsive element (TATC-box, GARE-motif and P-box) and the ABA-responsive element (ABRE) were found in the promoters of 12, 14, 25, 20 and 23 *CeqMYB* genes, respectively. In addition, stress related cis- elements including MBS (drought induced response element), LTR (low temperature response element), ARE (anaerobic induced response element), GC-motif (anoxic specific inducibility element) and TC-rich (defense and stress response element) were also identified in promoter regions of 32 selected *CeqMYB* genes. Therefore, the *CeqMYB* genes might be transcriptionally regulated under different abiotic stresses.

### Validation of *CeqMYB* genes expression following salt treatment

According with the expression pattern after salt treatment and GO annotation analysis, the expression of 32 selected *CeqMYB* genes in root and shoot were detected by qRT-PCR after different concentrations of NaCl were used to treat the clone A8 seedlings for different time points. In addition, the specific primers of qRT-PCR were listed in Table S9.

**Fig. 10:**
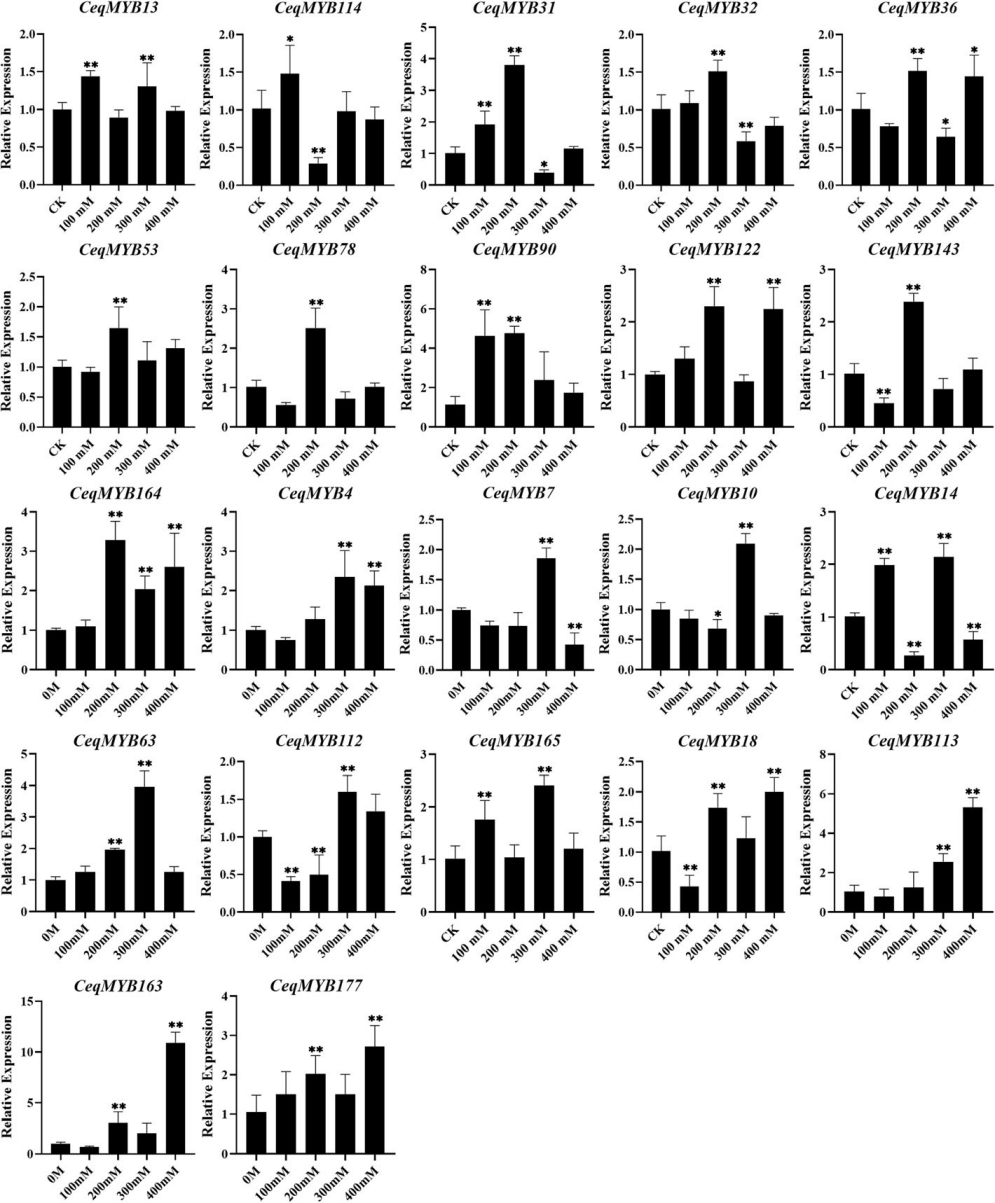
Relative expression of 22 *CeqMYB* genes following NaCl treatment at different concentrations in roots as determined by qRT-PCR. The Y-axis and X-axis indicated relative expression levels and salt concentration of stress treatment, respectively. Mean values and standard deviations (SDs) were obtained from three biological and three technical replicates. The error bars indicate standard deviation. **P < 0.01 and *P < 0.05

Fig. S1 showed that the expression of 90.6% (29/32) *MYB* genes was induced/repressed under different concentrations of NaCl treatment in *C. equisetifolia* roots. For example, expression of *CeqMYB18*, *CeqMYB113*, *CeqMYB163*, *CeqMYB177* were up-regulated and peaked at 400 mM. Furthermore, expression of 16 genes peaked at different concentrations (Fig. 10). For example, *CeqMYB31* and *CeqMYB90* were up-regulated under low salt treatment and reached the maximum at 200 mM, but then dropped subsequently. Moreover, *CeqMYB90* were significantly up-regulated more than 4-fold. In addition, the root response in salt stress at 200 mM NaCl shown in Fig. S2, all members of selected *CeqMYB*s can make relevant stress. Nine genes (*CeqMYB14*, *CeqMYB16*, *CeqMYB31*, *CeqMYB32*, *CeqMYB37*, *CeqMYB71*, *CeqMYB97*, *CeqMYB122* and *CeqMYB165*) were up-regulated at 24 h and 168 h, whereas only *CeqMYB164* was repressed with increasing time. Similar results were obtained in the paralogous (*CeqMYB113/− 163*), the expression levels were significantly up-regulated by high salinity treatment (400 mM) and at the time of 1 h in roots (Fig. 10 and Fig. S2).

**Fig. 11:**
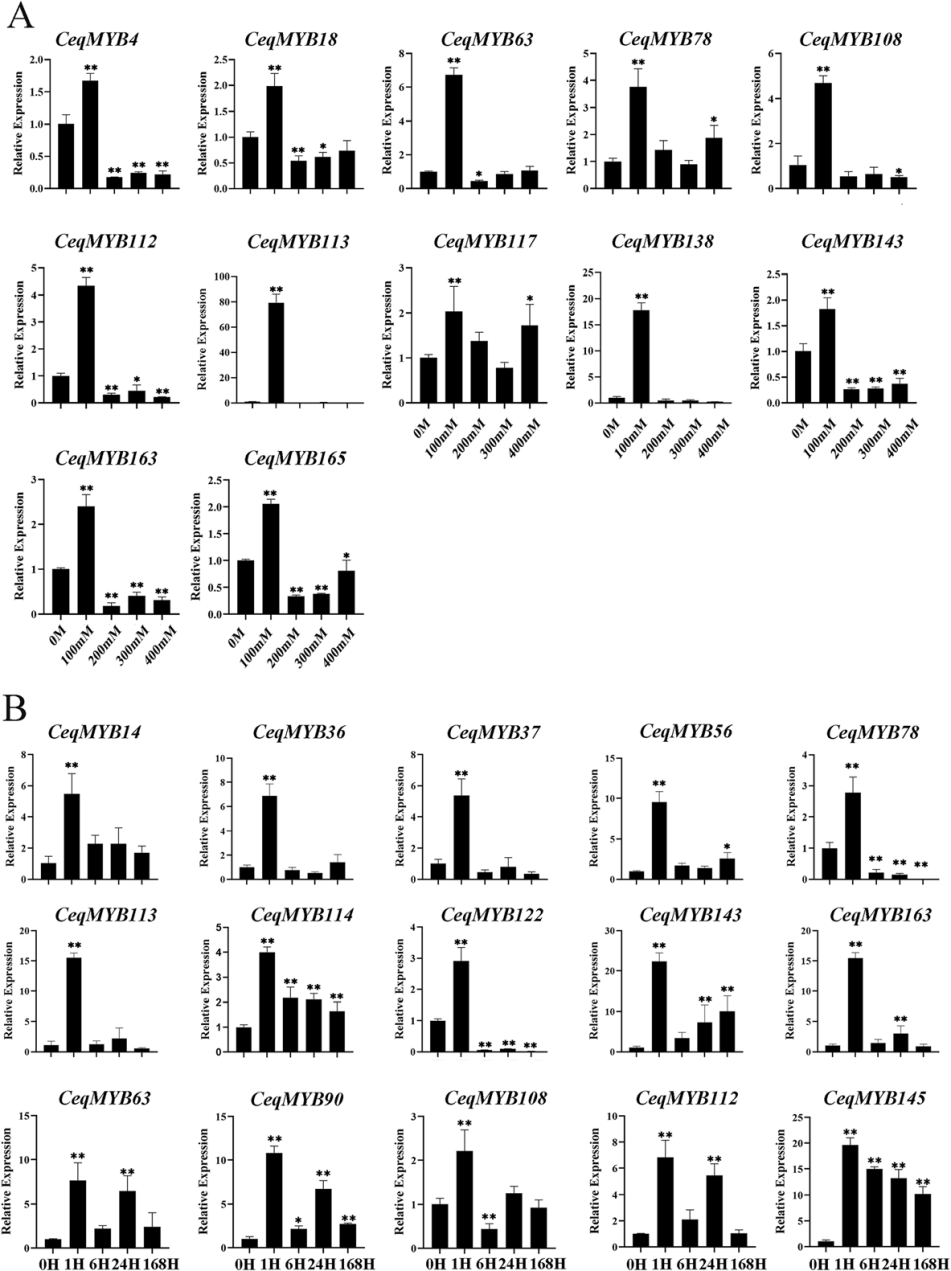
Relative expression of some *CeqMYB* genes following multiple NaCl treatment in roots as determined by qRT-PCR. **A** The expression level of 12 *CeqMYB* genes were up-regulated and peaked at 100 mM under different salt concentrations treatment. The Y-axis and X-axis indicated relative expression levels and salt concentration of stress treatment, respectively. **B** The expression level of 15 *CeqMYB* genes were up-regulated and peaked at 1 h following 200 mM NaCl treatment. The Y-axis and X-axis indicates relative expression levels and the time courses of stress treatments, respectively. Mean values and standard deviations (SDs) were obtained from three biological and three technical replicates. The error bars indicate standard deviation. **P < 0.01 and *P < 0.05

Next, we analyzed the *CeqMYB* genes expression profile in shoots after treating at different concentrations of NaCl and at the same concentration for different time. Of the 32 *CeqMYB* genes, 15 were up-regulated at different concentrations, while 16 genes were down-regulated, compared with untreated seedlings (Fig. S3). The 16 genes were intensely up-regulated and peaked at low salinity (100 mM), and were then distinctly down-regulated (Fig. 11A). *CeqMYB113* (more than 60-fold), *CeqMYB138* (more than 15-fold), *CeqMYB108* (more than 4-fold), *CeqMYB112* (more than 4-fold) and *CeqMYB63* (more than 6-fold) were strongly up-regulated in response to NaCl treatment at 100 mM. To further investigate the response of *CeqMYB* genes in shoots to salt stress, different time periods of salt treatment were used. From these, the expression level of *CeqMYB97* did not change significantly, 8 genes were induced and peaked at 24 h, and 3 genes were suppressed at different time points (Fig. S4). Furthermore, Fig. 11B shown that 15 of 32 *CeqMYB* genes were expressed and peaked at 1 h, indicating a possible role for these genes in immediate/early responses of seedlings to osmotic stress. *CeqMYB113* and *CeqMYB163* were typical of this trend, the expression of which remained relatively unchanged at later time points but was strongly up-regulated by about 15-fold at 1 h. In short, most *CeqMYB* genes were up-regulated in root and shoot by multiple NaCl treatment, suggesting these genes may play important roles in salt stress response.

## Discussion

*MYB* genes family is one of the largest transcription factors in plants. And *MYB* genes play important roles in many physiological processes of plants, such as cell cycle, primary metabolism, secondary metabolism, environmental response and stress response [4, 7, 38, 39]. So far, this is the first systematic study of the gene families in *C. equisetifolia* using bioinformatics tools and expression profiles based on the sequenced *C. equisetifolia* genome.

In this present study, a total of 182 *CeqMYB* genes were identified in the *C. equisetifolia* genome, and the distribution trend of *CeqMYB* members was similar to that reported in *A. thaliana* (Fig. 1B). The phylogenetic relationship of *MYB* gene family in *C. equisetifolia* and *A. thaliana* was studied (Figs. 1 and 2). Phylogenetic trees showed that some *MYB* genes in *C. equisetifolia* and *A. thaliana* formed their own independent clusters, which indicated that two species had a conserved evolutionary process. Moreover, not all groups of *CeqMYB* genes contain *AtMYB* genes. For example, the C4 group does not contain any *AtMYB*, reflecting the requirements of species to adapt to their specific environment [40].. The most closely related *MYB* genes may share a similar function. Previous studies have reported that *AtMYB44* enhanced transgenic soybean tolerance to drought/salt stress by positively regulating abscisic acid (ABA) signaling to induce stomatal closure [41], and *AtMYB73* played a negative role in plant tolerance to salt stress [27]. *AtMYB44* and *AtMYB73* were clustered in the same group, suggesting that *CeqMYB130/− 7/− 10/− 129/− 103* in C27 group responded to salt stress.

Furthermore, the conserved motif and gene structure of 182 *CeqMYB* genes were analyzed (Figs. 3 and 4). The number and distribution of conserved motifs and exon/intron structures were different in *CeqMYB* genes. Among them, 4 *CeqMYB* genes had only one motif, and the maximum had 10 motif elements. The motif distribution of the same group of *MYB* was relatively consistent, most motif elements were concentrated and regularly distributed at the N-terminal, and a few (such as motif 18 and motif 17 in R2R3-MYB) were irregularly distributed at the C-terminal, indicating that *CeqMYB* genes with a close evolutionary relationship had similar functions. Some motifs appeared more frequently, such as motif 4 and motif 2, reflecting their importance to the function of *CeqMYB* protein. In addition, the members of the *CeqMYB* genes were divided into the same groups with mostly similar gene structures. Combined with previous studies [42, 43], it was also found that the number of exons in most *MYB* genes did not exceed two introns. On the whole, the differences in the number, type and distribution of the conserved motifs and exon/intron structures in the sequences might reveal the different functions of each gene. Ka/Ks can reflect the selection pressure in the process of biological evolution. Ka/Ks analysis was performed on all the identified homologous pairs, and the results showed that all the Ka/Ks ratios were less than 1 (Fig. 6), indicating that the *MYB* gene was mainly affected by purifying selection during evolution (Table S6).

Many MYB proteins are involved in the response of plants to adverse growth environment, some of which were closely related to the regulation of plant salt stress. Recent research reported that *GhMYB108-like* would play important regulatory role in response to drought and salt stresses based on quantitative expression analysis [44] and overexpression of *TaMYB344* in tobacco enhanced the tolerance of plant to drought, high temperature and salt stress [45], while overexpression of *VcMYB4a* in blueberry callus enhanced sensitivity to salt, drought, cold, freezing, and heat stress [46]. Based on transcriptome data, we analyzed the expression patterns of 182 *CeqMYB* genes in response to 200 mM NaCl treatment at different time periods, and divided them into 25 groups according to different expression patterns (Fig. 7). Moreover, analysis of the molecular function annotations revealed that seven *CeqMYB* genes responded to salt stress (Fig. 8). From these, we selected 32 *MYB* genes to further explore their response to salt stress. Promoter analysis (Fig. 9) showed that 24 of the 32 selected *CeqMYB* genes harbored ABRE cis-regulatory elements involved in ABA responsiveness in their promoters. The auxin-responsive element (TGA-element and AuxRR-core) were also identified in the promoters of the 11 selected *CeqMYB* genes. Studies had shown that ABA signaling pathway played an important role in MYB-mediated salt tolerance. Overexpression of *TaMYB33* could enhance drought and salt tolerance in *Arabidopsis thaliana* through ABA-mediated regulation of stress response signals [47]. In addition, the R2R3-type MYB TF encoded by MULTIPASS (OSMPs) were involved in plant hormone and cell wall synthesis while responding to high salinity signals [48]. These results suggested that *MYB* TFs can enhance salt tolerance of plants by mediating the signaling of plant hormones.

And the elucidation of gene expression patterns can provide important clues about gene function. The expression levels of 32 *MYB* genes were examined in the root and shoot of *C. equisetifolia* under various NaCl concentrations and time points under salt stress. The expression of 22 genes in roots and 15 genes in shoots were up-regulated after different concentration of NaCl treatment (Fig. S1; Fig. S3). Specially, expression level of *CeqMYB16*, *CeqMYB37* and *CeqMYB56* did not change significantly in root, but down-regulate in shoots. And *CeqMYB71* were down-regulated at different concentration in root and shoot. Moreover, the expression level of some *CeqMYB* genes were up-regulated in root under salt stress, but down-regulated in shoots. The similar result was observed in *Helianthus annuus* [37], suggesting that some MYB genes were specifically expressed in roots. Some paralogous pairs exhibited similar expression patterns in the same tissue. For example, *CeqMYB7/− 10* peaked at 300 mM NaCl in roots, but dropped to less than half that of CK at different concentration of NaCl treatment in shoots. Most genes with the same number of exons had similar expression patterns, such as CeqMYB7/− 10 contained one exon and CeqMYB4/− 16/− 32 contained 3 three exons (Fig. S3). Furthermore, qRT-PCR experiments and RNA-Seq data analysis showed that most *CeqMYB* genes were up-regulated at different time points following salt treatment in roots and shoots (Fig. S2; Fig. S4), consistent with previous studies such as that reported by Zhou et al. who found that some *MYB* genes in oil palm were induced at 24 h or/and 48 h by 300 mM NaCl treatment [49]. At least 8 genes were expressed in peanut roots under salt stress by qRT-PCR analysis [50]. In addition, rapid induction of *AtMYB41* expression in response to osmotic and salt stress [51], and transgenic plants overexpressing *AtMYB74* displayed hypersensitivity to NaCl during seed germination [26]. *CeqMYB4*, *CeqMYB98*, *CeqMYB72*, *AtMYB41* and *AtMYB74* were clustered in the same group, which suggested that these three *CeqMYB* genes responded to salt stress. In this study, *CeqMYB4*, which was a homologous pair of *AtMYB74*, was strongly up-regulated by about 10-fold at 1 and 24 h. It once again showed that *CeqMYB4* played an important role in salt stress. Similarly, most *CeqMYB* genes were up-regulated to some extent following NaCl treatment, indicating a possible crucial role in response to salt stress.

## Conclusions

In this study, 182 *MYB* genes were identified in the genome of *C. equisetifolia*. A comprehensive bioinformatics analysis was performed to investigate phylogenetic relationships, conserved motifs, gene structure, and promoter analysis. GO annotation analysis of *CeqMYB* genes in *C. equisetifolia* revealed that seven *CeqMYB* genes were assigned to the “response to salt stress” category. Combined with expression profile analysis by RNA-Seq data, 32 *MYB* genes were selected to further explore their response to salt stress. Expression profiling of selected 32 genes in root and shoot were detected by qRT-PCR after different concentrations of NaCl were used to treat seedlings for different time point. The expression levels of most selected *MYB* genes were up-regulated in shoots and roots at different treatment. Moreover, the expression level of *CeqMYB4* was up-regulated under various salt treatments, indicating C*eqMYB4* might participated in the response to salt stress. The information provided by these results may be helpful for further functional analysis of *CeqMYB* gene to elucidate its salt stress mechanism in *C. equisetifolia*.

## Materials and methods

### Sequence retrieval and gene identification

The Casuarina genome data was downloaded from online website (http://forestry.fafu.edu.cn/db/Casuarinaceae/). *MYB* genes were identified according to previous research methods [3, 52]. The HMM profile of *MYB* TFs (PF00249) was downloaded from Pfam protein family database (http://pfam.xfam.org/), then used it as query (*P* < 0.001) for the identification of all putative *CeqMYB* genes. Finally, all candidate *MYB* genes were manually screened by the Pfam database (http://pfam.janelia.org/), the NCBI Conserved Domain database (http://www.ncbi.nlm.nih.gov/Structure/cdd/wrpsb.cgi) and the SMART database (http://smart.embl-heidelberg.de/). *A. thaliana* genome sequences were obtained from the Phytozome database (http://www.phytozome.net/). To analysis the number of amino acids, open reading frame (ORF) length, molecular weight (MW) and isoelectric point (pI) for each *MYB* gene by using ExPASy (http://www.expasy.ch/tools/pi_tool.html). In addition, through online website WoLP PSORT (https://wolfpsort.hgc.jp/), predict the subcellular localization of CeqMYB deduced proteins.

### Multiple sequence alignment and phylogenetic tree construction

MYB deduced proteins in *C. equisetifolia* were aligned with *AtMYB*s using ClustalX 2.11 [53, 54] software with default parameters. A neighbor-joining (NJ) phylogenetic analysis was conducted by MEGA7 based on the alignment. Bootstrap analysis with 1000 replicates was performed to calculate the reliability of the NJ tree [55].

### Exon–intron structural and conserved motif analysis

In order to map the gene structure of exon-intron distribution of MYB gene, the online Gene Structure Display Server (http://gsds.cbi.pku.edu.ch) was used. For this purpose, CDS of each MYB gene and its corresponding genomic DNA sequence need to be uploaded. The MEME online program (http://meme-suite.org/tools/meme) was employed to analysis conserved motifs of *MYB* superfamily members in *C. equisetifolia*. The parameters for performing this analysis were as follows: number of repetition = any; maximum number of motifs = 20; optimum motif length = 6–200 residues. And the each of the putative motifs was annotated by searching Pfam and SMART.

### Ka and Ks analysis of homologous pair

Based on the method from the study [56], defined paralogs. And the method was conducted by working a BLASTN [57] for all nucleotide sequences for each species. A pair of matching sequences that aligned exceed 300 bp and the identity ran over 80% were defined as pairs of paralogs in *C. equisetifolia*. The synonymous (Ks) and non-synonymous (Ka) substitutions per site between gene pairs were calculated by DnaSP v5.0 software [58].

### Gene ontology (GO) annotation analysis

Gene ontology (GO) analysis was carried out for the *CeqMYB* genes from agriGO database (http://systemsbiology.cau.edu.cn/agriGOv2/index.php). All the 23,397 genes of *C. equisetifolia* were taken as the reference set and *CeqMYB* genes for both samples were taken as the test set. The results were divided into three categories, namely molecular function, biological process, and cellular component.

### Expression profiling of *CeqMYB* genes

The expression profile data were downloaded from the Short Read Archive of the NCBI database (project accession number SRP064226) for expression analysis of *C. equisetifolia* root in different periods of salt treatment. The raw read counts for each transcript were calculated using Htseq-count and then normalized to transcripts per million (TPM). A heatmap was generated and visualized using the TBTOOLS software [59], the color scale shown represents TPM counts, and the ratios were log2 transformed. R software (https:// www.r-project.org) was used for the clustering analysis.

### Putative promoter Cis-acting element analysis

To further study the regulatory mechanism of the *CeqMYB* genes in the abiotic stress response, many cis-acting elements related to plant growth and development, phytohormonal response, and abiotic and biotic stress responses were identified through PlantCARE program. The upstream 1500 bp region of the translation start site of the *CeqMYB* genes were download from the *Casuarinaceae* Database. The PlantCARE program was used to screen cis-elements in putative. The elements involved in plant growth and development, hormone response, abiotic and biological stress response were summarized.

### Plant materials and salt treatments

The *C. equisetifolia* clone A8 (bred by the Zhanjiang Forestry Research Institute, selected from a commercial plantation in 1980s, which has been commercialized now, no any required permission for its sample collection and use) was preserved and cultivated by the Research Institute of Tropical Forestry, Chinese Academy of Forestry. Rooted cuttings of the clone A8 cultured in a growth chamber for 3 months were prepared for the experiment. For salt treatments, roots and shoots were harvested at 0, 1, 6, 24 and 168 h after 200 mM NaCl treatment, respectively. Furthermore, various concentrations (0, 100, 200, 300 and 400 mM NaCl) solution was poured over the culture medium vermiculite and black soil. The roots and shoots were harvested following 24 h of salt treatment and immediately frozen in liquid nitrogen, and transferred to an ultra-low temperature freezer for storage at-80 °C prior to needed for RNA extraction.

### RNA extraction and qRT-PCR analysis

Total RNA was extracted from roots using the Aidlab plant RNA kit (Aidlab Biotech, Beijing, China) based on specifications. The integrity and concentration of the RNA was verified by 1% agarose gel electrophoresis and NanoDrop™ One/OneC (ThermoFisher Scientific, USA). The first strand cDNA was synthesized by PrimerScript RT MasterMix (Takara, Tokyo, Japan) according to the manufacturer’s instructions. qRT-PCR was performed on an LightCycler480 II Real-Time PCR system (Made in Switzerland) using TB Green Premix Ex Taq II (TaKaRa Biotechnology Co. Ltd., Dalian, China) with a 20 μL sample volume. And each reaction mixture contained 2.0 μl of diluted cDNA, 0.8 μl of each primer, 10.0 μl of TB Green Premix Ex Taq II, and 6.4 μl of RNase-free water. qPCR reaction cycling conditions were set as per the manufacturer’s instructions for TB Green Premix Ex Taq II. Each sample was conducted three times biologically using replicate. The relative expression level of each gene was calculated as 2^-ΔΔCT^ [60] compared with untreated control plants that were set as 1. Specific primers for *CeqMYB* genes were designed by Primer Premier 5.0 software and the *EF1α* was used as housekeeping gene [61]. Statistical analysis and drawing by GraphPad 8 software [62].

### Statistical analysis

Statistical significance was performed using a paired Student’s t test by JMP 8 software. The mean values and standard deviations (SDs) were calculated from three biological and three technical replicates, and significant differences relative to controls are indicated at **P < 0.01 and *P < 0.05.

## Supplementary Information

**Additional file 1: Figure S1**. Relative expression of 32 selected *CeqMYB* genes following NaCl treatment at different concentrations in roots by qRT-PCR. The Y-axis and X-axis indicated relative expression levels and salt concentration of stress treatment, respectively. Mean values and standard deviations (SDs) were obtained from three biological and three technical replicates. The error bars indicate standard deviation. **P < 0.01 and *P < 0.05.

**Additional file 2: Figure S2**. Relative expression of 32 selected *CeqMYB* genes following NaCl treatment at different time periods in roots by RNA-Seq. The heatmap shows the hierarchical clustering of 32 *CeqMYB* genes at different time points. The color scale represents log10 expression values, blue represents low expression and red indicates a high expression level (transcript abundance).

**Additional file 3: Figure S3**. Relative expression of 32 selected *CeqMYB* genes following NaCl treatment at different concentrations in shoots by qRT-PCR. The Y-axis and X-axis indicated relative expression levels and salt concentration of stress treatment, respectively. Mean values and standard deviations (SDs) were obtained from three biological and three technical replicates. The error bars indicate standard deviation. **P < 0.01 and *P < 0.05.

**Additional file 4: Figure S4**. Relative expression of 32 selected *CeqMYB* genes following NaCl treatment at different time periods in shoots by qRT-PCR. The Y-axis and X-axis indicates relative expression levels and the time courses of stress treatments, respectively. Mean values and standard deviations (SDs) were obtained from three biological and three technical replicates. The error bars indicate standard deviation.

**Additional file 5: Table S1**. Details of the identified *CeqMYB* genes. **Table S2** The prediction of subcellular localization in *CeqMYB* genes. **Table S3** Putative functions of the MYB proteins in *Casuarina equisetifolia*. **Table S4** Detailed information for the 20 motifs in the R2R3-MYB proteins of *Casuarina equisetifolia*. **Table S5** Detailed information for the 20 motifs in the 1R-MYB, 3R-MYB and 4R-MYB proteins of *Casuarina equisetifolia*. **Table S6** Ka/Ks ratios of gene pairs in *Casuarina equisetifolia*. **Table S7** Heat map data of 182 MYB gene family in *Casuarina equisetifolia*. **Table S8** Gene ontology of the of MYB gene family in *Casuarina equisetifolia*. **Table S9** List of primer sequences used for qRT-PCR analysis of 32 selected *CeqMYB* genes.

## Data Availability

Raw Illumina sequence data were deposited in the Short Read Archive of the NCBI database (project accession number SRP064226). The datasets supporting the results of this article are included in the article and Additional files.
