# Supplementary figures and images for: Genome-wide analysis of MYB transcription factors and their responses to salt stress in Casuarina equisetifolia
Source: BMC Plant Biol. 2021 Jul 8;21:328. doi: 10.1186/s12870-021-03083-6 (PMC8265015; doi:10.1186/s12870-021-03083-6)

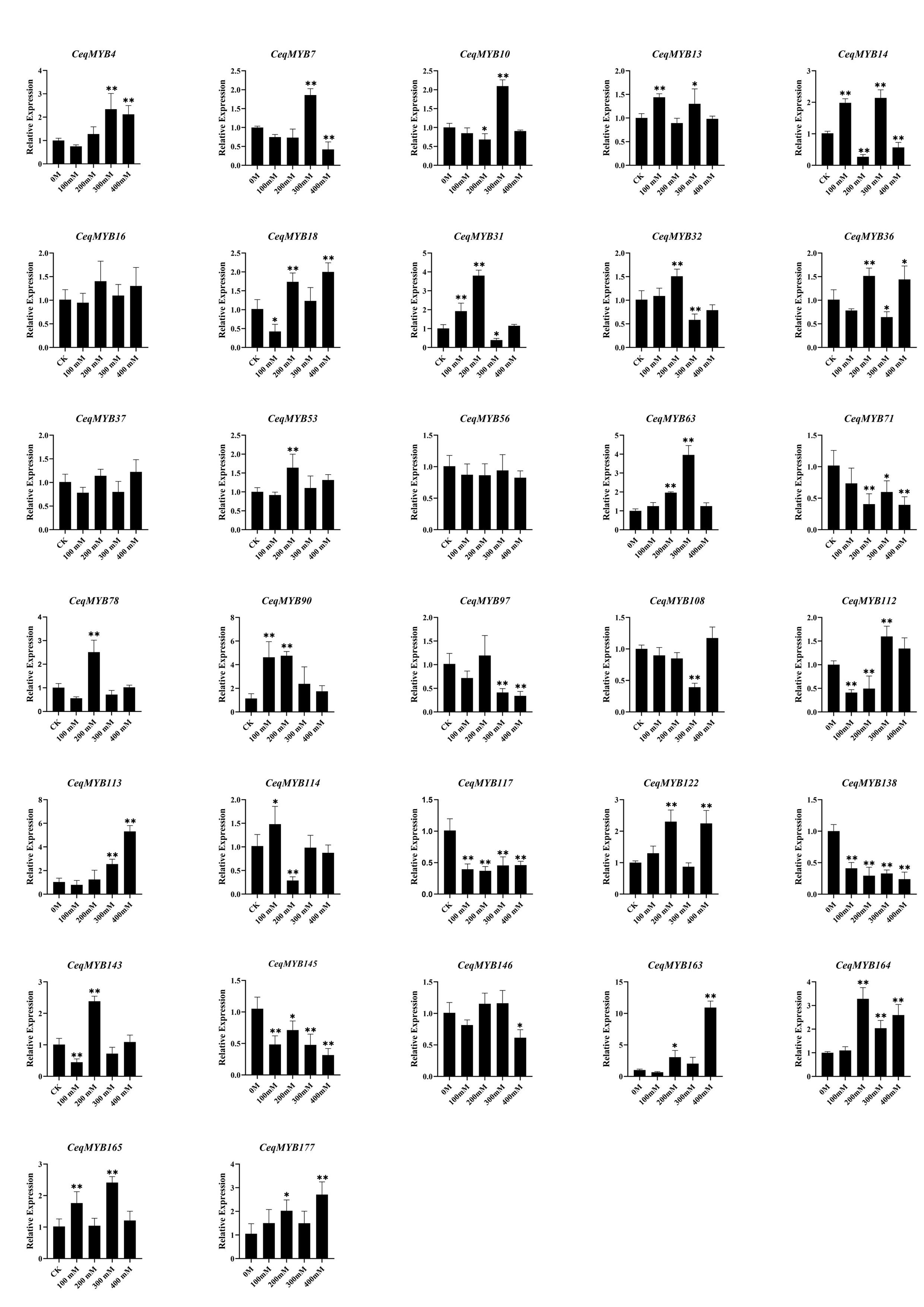

Supplement: Supplementary file 1 — Additional file 1: Figure S1. Relative expression of 32 selected CeqMYB genes following NaCl treatment at different concentrations in roots by qRT-PCR. The Y-axis and X-axis indicated relative expression levels and salt concentration of stress treatment, respectively. Mean values and standard deviations (SDs) were obtained from three biological and three technical replicates. The error bars indicate standard deviation. **P < 0.01 and *P < 0.05. [file 12870_2021_3083_MOESM1_ESM.tif]

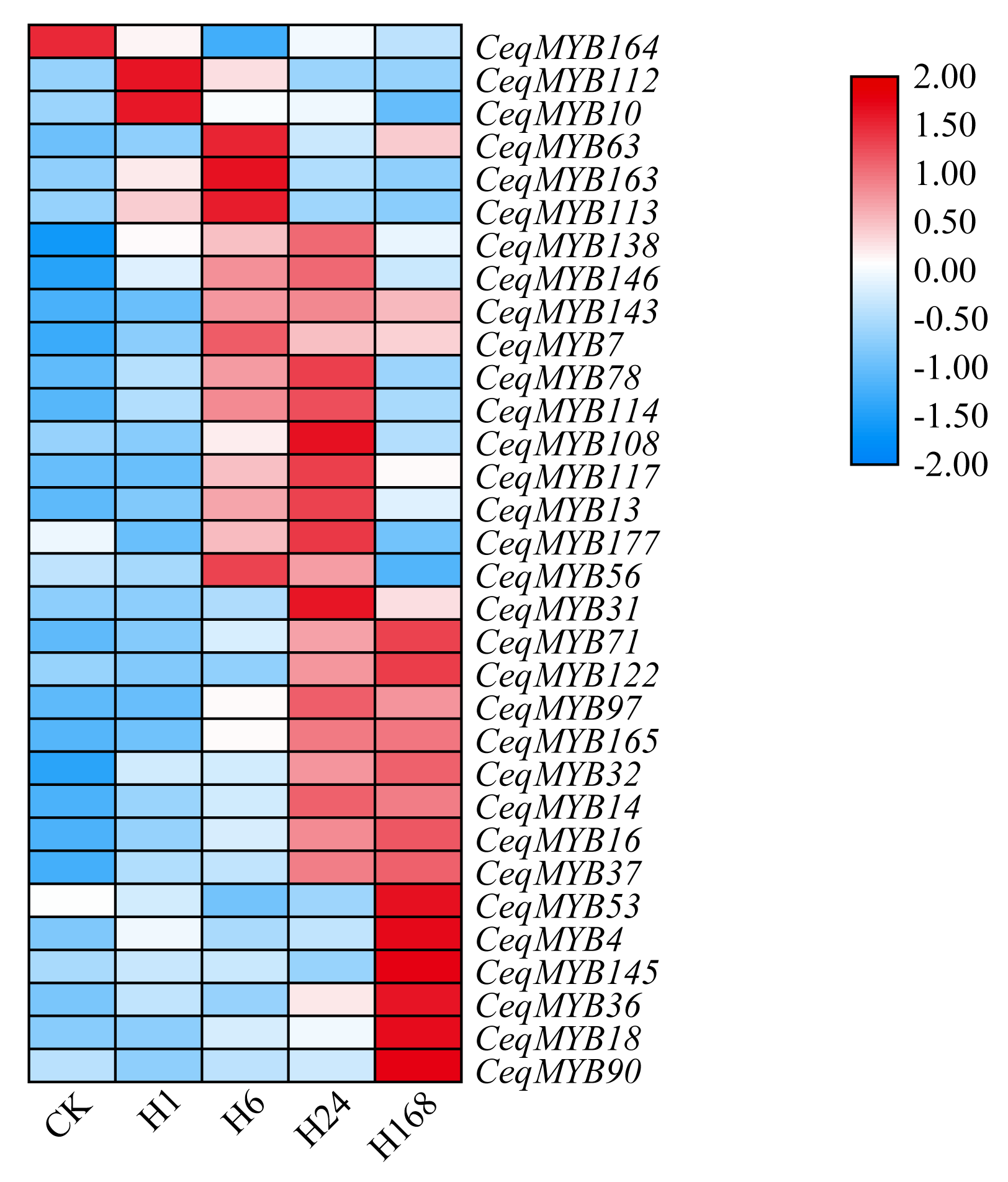

Supplement: Supplementary file 2 — Additional file 2: Figure S2. Relative expression of 32 selected CeqMYB genes following NaCl treatment at different time periods in roots by RNA-Seq. The heatmap shows the hierarchical clustering of 32 CeqMYB genes at different time points. The color scale represents log10 expression values, blue represents low expression and red indicates a high expression level (transcript abundance). [file 12870_2021_3083_MOESM2_ESM.tif]

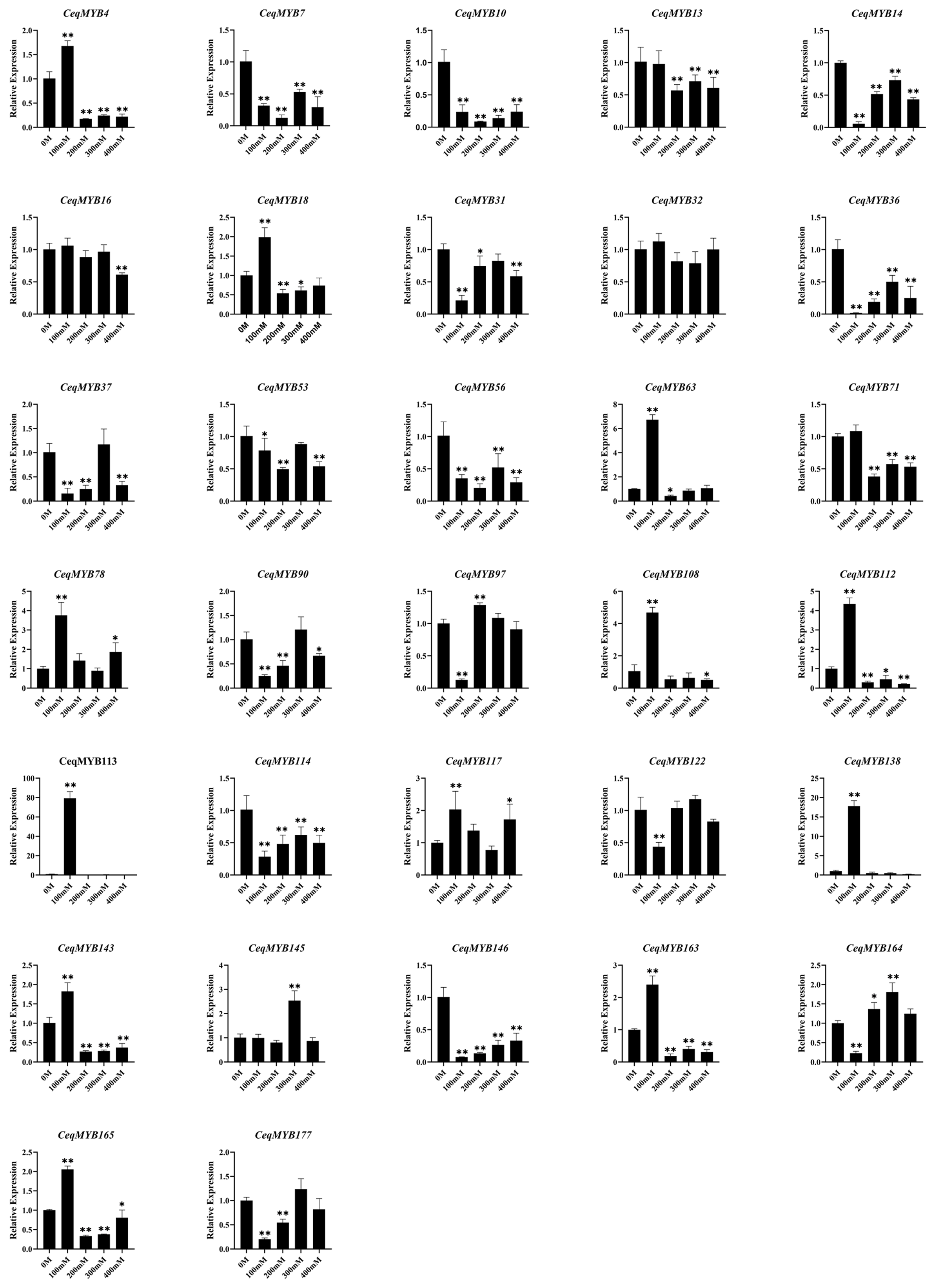

Supplement: Supplementary file 3 — Additional file 3: Figure S3. Relative expression of 32 selected CeqMYB genes following NaCl treatment at different concentrations in shoots by qRT-PCR. The Y-axis and X-axis indicated relative expression levels and salt concentration of stress treatment, respectively. Mean values and standard deviations (SDs) were obtained from three biological and three technical replicates. The error bars indicate standard deviation. **P < 0.01 and *P < 0.05. [file 12870_2021_3083_MOESM3_ESM.tif]

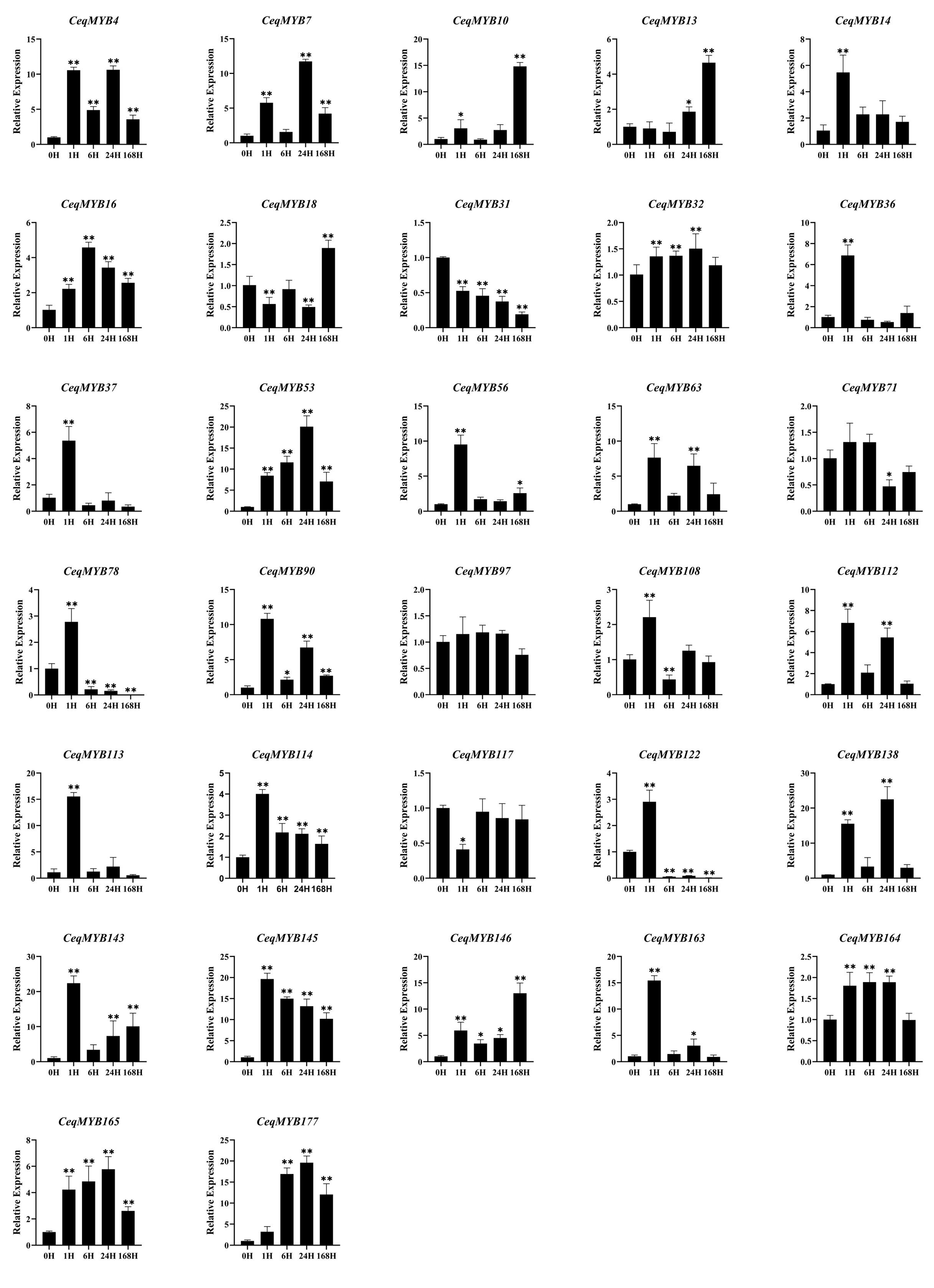

Supplement: Supplementary file 4 — Additional file 4: Figure S4. Relative expression of 32 selected CeqMYB genes following NaCl treatment at different time periods in shoots by qRT-PCR. The Y-axis and X-axis indicates relative expression levels and the time courses of stress treatments, respectively. Mean values and standard deviations (SDs) were obtained from three biological and three technical replicates. The error bars indicate standard deviation. [file 12870_2021_3083_MOESM4_ESM.tif]
